# Predictors of Diastolic-To-Wedge Gradient in Patients Evaluated for Pulmonary Hypertension

**DOI:** 10.1371/journal.pone.0076461

**Published:** 2013-10-04

**Authors:** Evan L. Brittain, Meredith E. Pugh, Li Wang, Alex L. Newman, Ivan M. Robbins, John H. Newman, Anna R. Hemnes

**Affiliations:** 1 Division of Cardiovascular Medicine, Vanderbilt University Medical Center, Nashville, Tennessee, United States of America; 2 Division of Pulmonary and Critical Care Medicine, Vanderbilt University Medical Center, Nashville, Tennessee, United States of America; 3 Department of Biostatistics, Vanderbilt University Medical Center, Nashville, Tennessee, United States of America; Keio University School of Medicine, Japan

## Abstract

**Background:**

Differentiation of pulmonary arterial hypertension (PAH) and pulmonary venous hypertension (PVH) often requires right heart catheterization (RHC). We sought to determine whether a combination of clinical and echocardiographic variables could predict the pulmonary diastolic to wedge (PAd-PWP) gradient and thus differentiate patients with PAH and PVH.

**Methods:**

We prospectively enrolled 108 patients presenting for PH evaluation. We developed a multivariate model to predict PAd-PWP gradient and validated this model using bootstrapping technique.

**Results:**

PAH patients had worse hemodynamics and were more likely to have evidence of right ventricular dilation and dysfunction whereas patients with PVH were older and more likely to have features of the metabolic syndrome. PAd-PWP gradient of ≥ 6mmHg accurately discriminated patients with PAH compared to PVH. Our model including clinical and echocardiographic variables was highly accurate for the prediction of PAd-PWP gradient with a slope 0.89 (slope of 1 represents perfect prediction).

**Conclusions:**

In this prospective study of patients referred for PH evaluation, a model of readily available clinical parameters and simple echocardiographic measurements accurately predicted the PAd-PWP gradient, allowing discrimination of patients with PAH and PVH. This model requires validation in a larger cohort, but may afford clinicians more parsimony with referral for invasive testing in the evaluation of PH.

## Introduction

Pulmonary arterial hypertension (PAH) is characterized by progressive obliteration of the pulmonary arterioles, elevated pulmonary vascular resistance (PVR) and eventual right heart failure. PAH confers a poor prognosis compared to other causes of pulmonary hypertension (PH), but is often difficult to differentiate from other etiologies [1–3]. Echocardiography or clinical characteristics alone are often inadequate to differentiate pulmonary venous hypertension (PVH) and PAH [4,5]. The distinction is critical as PAH–specific therapy may be deleterious to patients with PVH [6,7]. Thus, right heart catheterization (RHC) is required to differentiate PAH from PVH. Better bedside tools are needed to identify patients at low risk for PAH and potentially negate the need for invasive evaluation.

There are limited data on how to differentiation of PAH from PVH clinically, with most prior literature focusing on the hemodynamic differences [8]. Echocardiography is frequently able to differentiate patients with PAH and PVH with structural evidence of left heart disease such as left ventricular or left atrial enlargement, but fails to adequately classify the majority of patients [9]. Moreover, many patients undergo right heart catheterization to evaluate apparent modest elevations in echocardiographically estimated right ventricular systolic pressure, but are not found to have PH. The ideal clinical risk assessment would also address patients who are evaluated for dyspnea and found to have a mild increase in right ventricular systolic pressure on echocardiography.

The transpulmonary gradient (TPG; mean pulmonary artery pressure-pulmonary artery wedge pressure [PWP]), is thought to be a reflection of the underlying etiology of PH; TPG is low in PVH due to passive upstream transmission of elevated left-sided pressure and high in PAH due to pre-capillary vasoconstriction or structural changes. However, TPG is influenced by stroke volume, arterial compliance, and elevated left atrial pressure (LAP) and may therefore misclassify the etiology of PH in some patients [10,11]. The pulmonary artery diastolic (PAd) to pulmonary wedge pressure gradient (PAd-PWP) is less subject to changes in pulmonary flow and may therefore be useful in identification of patients in whom PH is predominantly a passive process or who have normal pulmonary hemodynamics [10]. Prediction of low PAd-PWP in patients with no specific risk factors for PAH may allow parsimony in the referral for invasive hemodynamics and direct clinicians’ attention to the investigation and treatment of underlying causes of PVH. The ability of non-invasive variables, including clinical and echocardiographic characteristics, to predict the PAd-PWP gradient has not been studied.

We hypothesized that a combination of clinical and simple echocardiography-based variables could be used to predict the PAd-PWP gradient. We used an existing prospectively collected dataset to develop and internally validate a statistical model using these variables to predict hemodynamic differences in PAH and PVH and patients with no pulmonary hypertension.

## Methods

### Ethics Statement

This prospective, single-center study was approved by the Vanderbilt Institutional Review Board and all subjects signed informed consent.

### Clinical Setting and Patients

All new or returning patients aged >18 years who were evaluated in the Vanderbilt University Center for Pulmonary Vascular Disease from August 2009 through March 2010 were eligible for enrollment. Right heart catheterization was performed by either of two cardiologists using a standard protocol. PH was defined as mean pulmonary artery pressure (mPAP) of ≥ 25 mm Hg by cardiac catheterization. PAH required a pulmonary artery wedge pressure (PWP) measured at end-expiration of ≤ 15 mm Hg [12]. Patients with PVH had a PWP > 15 mm Hg at rest, or increased PWP > 7 mm Hg after infusion of 1L of normal saline to a value > 15mmHg [13,14]. Rapid infusion of 1L saline was recently shown to increase PWP approximately 6mmHg in health subjects [15]. Fluid challenge was performed over five minutes in all patients unless right atrial pressure or PWP was > 15mmHg. Exclusion criteria [2] were ≥ 5 L/min nasal cannula oxygen, portopulmonary hypertension (due to cirrhosis-associated hyperventilation), serum bicarbonate level of > 34 mmol/L, pregnancy, known neuromuscular disease, moderate or severe mitral stenosis, mitral or aortic regurgitation, left ventricular ejection fraction <55% by echocardiography, known hypercarbic respiratory failure, untreated hypothyroidism or hyperthyroidism, hereditary hemorrhagic telangiectasia, uncertain diagnosis because of incomplete testing, diagnosis of World Health Organization group 3 or 4 PH, or mixed PH phenotype after thorough evaluation according to published guidelines [12].

### Measurements

Demographic data, results of blood tests, 6MWT, and pulmonary function testing were extracted from the medical record. RHC data were manually reviewed by two authors with extensive experience in the evaluation and management of PH (I.M.R. and A.R.H.). The 6MWTs were performed according to American Thoracic Society criteria [16], although patients were not required to perform 6MWT for study enrollment. Echocardiograms were reviewed by an experienced echocardiographer (E.B.) blinded to patient diagnosis. RV dilation was defined as basal dimension > 42mm and mid-ventricular dimension > 35mm in a right ventricle-focused apical four-chamber view [17]. RV hypertrophy was defined as RV wall thickness > 5mm in either the subcostal or parasternal long-axis view [17]. Presence of RV dysfunction and paradoxical septal motion were based on visual assessment. RHC and echocardiography were performed as part of a routine evaluation of pulmonary hypertension. Right ventricular hypertrophy on electrocardiogaphy was defined as a ratio of R and S in lead V1 > 1 [18].

### Clinical and Echocardiographic Variables

We pre-specified several clinical variables which would indicate a likely pulmonary venous etiology of PH: history of coronary artery disease (CAD), prior cardiac surgery, history of percutaneous coronary intervention (PCI), history of atrial fibrillation (AF) or flutter, and prior AF ablation. In addition, we pre-specified several echocardiographic variables which would indicate likely PVH (left atrial diameter > 4.0 cm) or PAH (evidence of RV dysfunction, RV dilation, RV hypertrophy (RVH), or paradoxical septal motion). We also pre-specified several risk factors (age, sex, body mass index [BMI], diastolic blood pressure, diabetes mellitus [DM], hypertension [HTN], beta blocker use and RVH on electrocardiography [EKG]) to be included in the model in conjunction with the echocardiographic variables, clinical variables, or both (Table **1**).

**Table 1:** Risk Factors, Clinical and Echocardiographic Variables Used in Prediction Model.

**Risk Factors**	**Clinical Variables**	**Echo Variables**
Age	CAD	RV dysfunction
Sex	Prior cardiac surgery	RV dilation
Body mass index	Prior of PCI	RV hypertrophy
Diastolic blood pressure	Atrial fibrillation or flutter	Paradoxical septal motion
Diabetes mellitus	Prior atrial ablation	Left Atrial Enlargement
Hypertension		
Beta-blocker use		
RVH on EKG		

EKG = electrocardiogram; PCI = percutaneous coronary intervention; RV = right ventricular; RVH = right ventricular hypertrophy

### Statistical Considerations

Descriptive statistics were presented as mean ± standard deviation, median (interquartile range [IQR]), or percentage (N) as appropriate. Continuous variables were compared between groups using Wilcoxon rank sum test and Person’s chi-squared test for categorical variables. Clinical variables and echocardiographic variables which indicate the same clinical aspects were grouped into clusters. We used linear model with least squares to assess the association between PAd-PWP and the clinical/echocardiographic variables with adjustment of age, sex, BMI, diastolic blood pressure, DM, HTN, beta blocker use and RVH on EKG. All the continuous variables were modeled as linear trend due to the limited sample size. To test for a difference in the predictive value of clinical factors and echocardiographic factors, we used the LR χ **²** test for nested models to assess whether echocardiographic variables add predictive value to a model that includes clinical variables and whether clinical variables adds predictive value to a model that includes echocardiographic variables. The adequacy index is the fraction of the total LR χ **²** explained by a set of variables that could be explained by omitting the competing variables. The prediction model was internally validated and calibrated using bootstrapping technique with 300 bootstrap resamples. Model validation estimates the model’s future performance. Model calibration estimates the accuracy of the model’s predicted outcome compared to the observed outcome, which can be demonstrated using a smooth nonparametric calibration curve or plot of predicted versus observed outcome. Bootstrap samples were created by drawing random samples with replacement from the original sample with same size. A model was derived in each bootstrap sample and was applied without change to the original sample. Optimism was then calculated as the accuracy index from the bootstrap sample minus the index computed on the original sample. This process was repeated for 300 times and the average optimism was used to obtain the overfitting-corrected estimate.

All analyses were done with the statistical programming language R, version 2.15.1 (R Development Core Team, Vienna, Austria). The level of statistical significance was set at p<0.05.

## Results

### Study Patients

The composition of the study population has been previously reported [2]. For this analysis, we included 85 patients with PAH, 16 with PVH and 7 patients with no evidence of pulmonary hypertension and only mild or moderate pulmonary function testing abnormalities (NoPH). Demographic and clinical characteristics of the PAH and PVH groups are detailed in Table **2**. Of the 85 patients with PAH, 17 were functional class 1, 41 were functional class 2, 26 were functional class 3, and one was functional class 4. Causes for PAH were idiopathic (n = 33), connective tissue disease (n = 24), heritable PAH (n = 7), congenital heart disease (n = 15), and miscellaneous (n = 6).

**Table 2:** Demographic and Clinical Characteristics.

	**PAH (n=85)**	**PVH (n=16)**	**p Value**
**Age (years)**	50.9 ± 14.3	63.9 ± 12.2	0.001
**Number Female (%)**	63 (74)	12 (75)	0.74
**BMI**	28.6 ± 6.4	34.5 ± 8.2	0.002
**Comorbid Conditions**			
**Diabetes Mellitus, No (%)**	12 (14)	11 (69)	< 0.001
**Systemic Hypertension, No (%)**	26 (31)	13 (81)	< 0.001
**Hyperlipidemia, No (%)**	22 (26)	12 (16)	0.002
**CAD**	7 (8)	7 (44)	<0.001
**Prior Cardiac Surgery**	2 (2)	1 (6)	0.43
**PCI**	5 (6)	0 (0)	0.59
**Atrial Fibrillation**	2 (2)	5 (31)	0.001
**Atrial Fibrillation Ablation**	2 (2)	5 (31)	0.001
**Six Minute Walk Distance (m**)	369 ± 115	300 ± 118	0.04
**Medications**			
**Beta-blocker**	10 (12)	11 (69)	< 0.001
**ACEI or ARB**	12 (14)	6 (38)	0.03
**Resting Heart Rate (beats per minute)**	86 ± 14	71 ± 12	< 0.001
**Heart Rate after Six Minute Walk (beats per minute)**	112 ± 20	93 ± 21	0.003
**Pulmonary Function Test**			
**DLCO (% predicted)**	63 ± 24	76 ± 21	0.05
**FEV1 (% predicted)**	73 ± 17	71 ± 16	0.52
**FVC (% predicted)**	71 ± 17	72 ± 15	0.14
**Plasma Bicarbonate (mmol/L)** **RVH on EKG**	25.7 ± 3.2 39 (57)	28.1 ± 2.4 0 (0)	0.006 < 0.001
**Functional Class, No (%)**			0.04
**I**	17 (20)	0 (0)	
**II**	41 (48)	6 (38)	
**III**	25 (29)	11 (69)	
**IV**	1 (1)	0 (0)	

ACEI/ARB = angiotensin converting enzyme inhibitor or angiotensin receptor blocker; BMI = body mass index; CAD = coronary artery disease; DLCO = diffusion capacity of the lung for carbon monoxide; EKG = electrocardiography; FEV1 = forced expiratory volume in 1 second; FVC = forced vital capacity; PCI = percutaneous coronary intervention; RVH = right ventricular hypertrophy

As shown in Table 2, patients with PVH were more likely to have comorbidities related to cardiovascular and metabolic disease. PVH patients were also more likely to be on beta-blocker or ACEI/ARB therapy which reflects an increased prevalence of systemic hypertension and atrial fibrillation in that cohort. Over half of the PAH patients had evidence of RVH on electrocardiography compared to none of the patients with PVH.

### Echocardiography in PAH and PVH

In order to allow our prediction model to be easily integrated into clinical practice we chose echocardiographic parameters easily assessed on any patient with adequate image quality and excluded highly quantitative, load-dependent, or Doppler-based parameters. Echocardiographic variables for PAH and PVH patients are shown in Table **3**. The presence of each variable was more frequent in PAH compared to PVH (p < 0.05 for all). Only one patient with NoPH had RV dilation and none had RV dysfunction, RV hypertrophy, or paradoxical septal motion.

**Table 3:** Echocardiographic Features.

	**PAH (n = 82)**	**PVH (n = 16)**	**p value**
**RV Dysfunction (%)**	56 (68)	6 (38)	0.02
**RV Dilation (%)**	71 (87)	7 (44)	< 0.001
**RV Hypertrophy (%)**	38 (48)	1 (6)	0.005
**Paradoxical Septal Motion (%)**	48 (59)	2 (13)	< 0.001
**LA Diameter > 4.0cm (%)**	23 (29)	9 (56)	0.04

LA = left atrium; RV = right ventricle

### Hemodynamics in PAH and PVH

**Figure 1:**
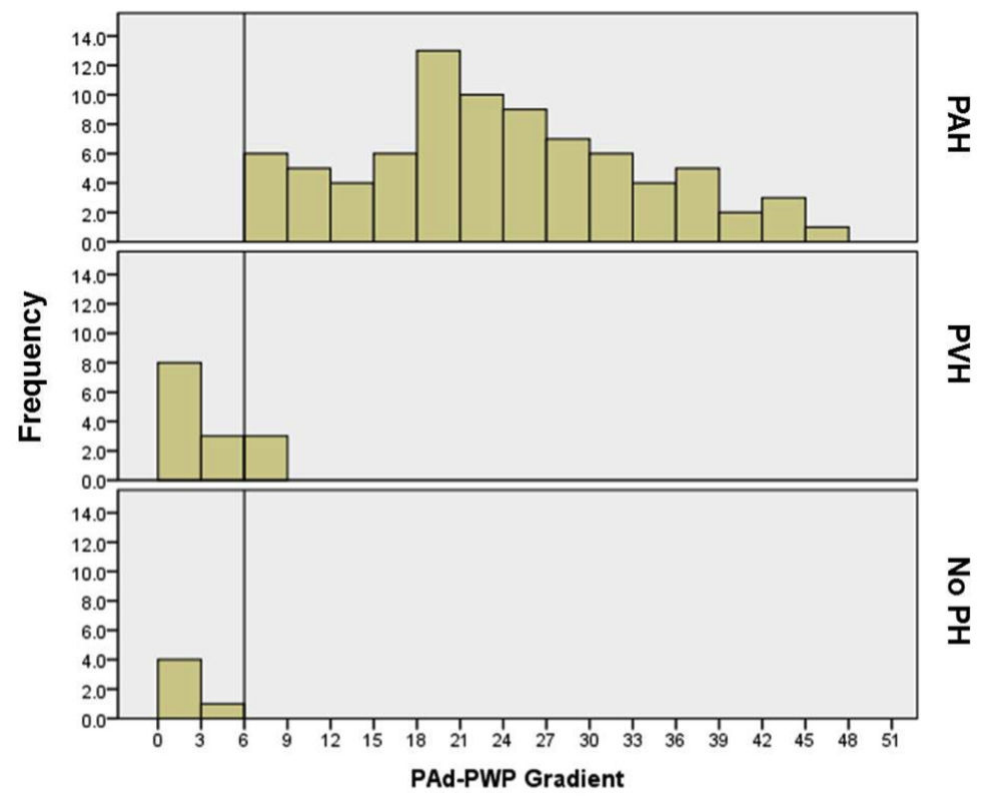
Distribution of Pulmonary Artery Diastolic to Wedge Gradient in Patients with PAH, PVH, and No PH. A PAd-PWP gradient of 6 accurately discriminates patients with PAH and PVH. The two patients with PVH had a PAd-PWP of 7mmHg both of whom had very elevated PWP. No patients with PAH had a PAd-PWP <7mmHg. No PH = no evidence of PH on invasive measurement; PAd-PWP = pulmonary artery diastolic to wedge gradient; PAH = pulmonary arterial hypertension; PVH = pulmonary venous hypertension.

Detailed hemodynamic data for the PAH and PVH groups are shown in Table **4**. Both groups had elevated mean PA (mPA) pressure but left atrial pressure was significantly higher in the PVH group. The PAd-PWP gradient was significantly higher in the PAH group reflecting elevated vascular resistance at the level of the pulmonary arterioles (24.0 ± 11.8mmHg vs. 2.7 ± 2.8 mmHg in PVH). A cutoff of 6mmHg for PAd-PWP gradient accurately stratified patients with PAH and PVH (Figure **1**). Two patients with PVH had a PAd-PWP gradient of 6-7mmHg; however these patients had PWPs of 24 and 26mmHg and were thus easily identified as having PVH. The 7 patients with NoPH had a mPA pressure of 17.6 ± 5.2mmHg, PWP of 9.4 ± 2.4mmHg, and PVR of 1.4 ± 1.1WU.

**Table 4:** Hemodynamic Data.

	**PAH (n = 85)**	**PVH (n = 16)**	**p value**
**Right atrial pressure (mmHg)**	7.6 ± 5.8	11.8 ± 5.5	0.007
**Mean pulmonary artery pressure (mmHg)**	50.8 ± 14.7	35.9 ± 11.1	0.001
**Pulmonary wedge pressure (mmHg)**	9.0 ± 4.6	20.2 ± 6.1	< 0.001
**Cardiac index (l/m/m^2^)**	2.6 ± 0.9	3.4 ± 0.8	0.001
**Pulmonary vascular resistance (Wood units)**	9.3 ± 5.0	2.9 ± 1.8	< 0.001
**Transpulmonary gradient (mmHg)**	41.9 ± 14.3	15.7 ± 7.5	< 0.001
**Pulmonary artery diastolic to wedge gradient (mmHg)**	24.3 ± 11.4	2.7 ± 2.8	< 0.001

### Clinical and Echocardiographic Variables

To study the ability of the pre-specified clinical and echocardiographic variables to predict PAd-PWP, we fit three linear models: clinical variables, echocardiographic variables, and combined. All 3 models included an identical set of other risk factors, which were: age, sex, BMI, diastolic blood pressure, diabetes mellitus (DM), hypertension (HTN), beta-blocker use, and RVH on electrocardiography. Using the LR χ **²** test for nested models, clinical factors did not add predictive value to a model that includes echocardiographic factors (LR χ **²** =0.008, df=1, p=0.928), whereas echocardiographic factors added clinically and statistically significant predictive value to a model that includes clinical factors (LR χ **²** = 10.214, df=1, p=0.001). The adequacy index for clinical factors model and echocardiographic factors model were 0.802 and 1, respectively. The influence of each of the risk factors and echocardiographic variables in the final model on the predicted PAd-PWP is shown Table **5** with effect indicating the magnitude and direction of each variable on Pad-PWP.

**Table 5:** Multivariable Linear Model Predicting Pulmonary Artery Diastolic to Wedge Gradient.

	**Effect**	**95% CI**	**P-value**
**Age (per 5 years)**	-0.29	(-1.17-0.6)	0.52
**Sex (female)**	4.33	(-1.44-10.09)	0.15
**BMI (per 5kg/m^2^)**	1.79	(-0.1-3.69)	0.06
**Diastolic BP (per 10mmHg)**	1.32	(-0.64-3.28)	0.18
**Diabetes (present)**	-5.94	(-11.42--0.46)	0.04
**Hypertension (present)**	-6.0	(-11.52--0.47)	0.04
**Beta blocker use (yes)**	-1.89	(-7.71-3.93)	0.53
**RVH on EKG (present)**	4.85	(-0.07-9.77)	0.06
**Echocardiographic variable (per 1 present)**	2.84	(1-4.68)	0.003

BMI = body mass index; BP = blood pressure; EKG = electrocardiogram; RVH = right ventricular hypertrophy

**Figure 2:**
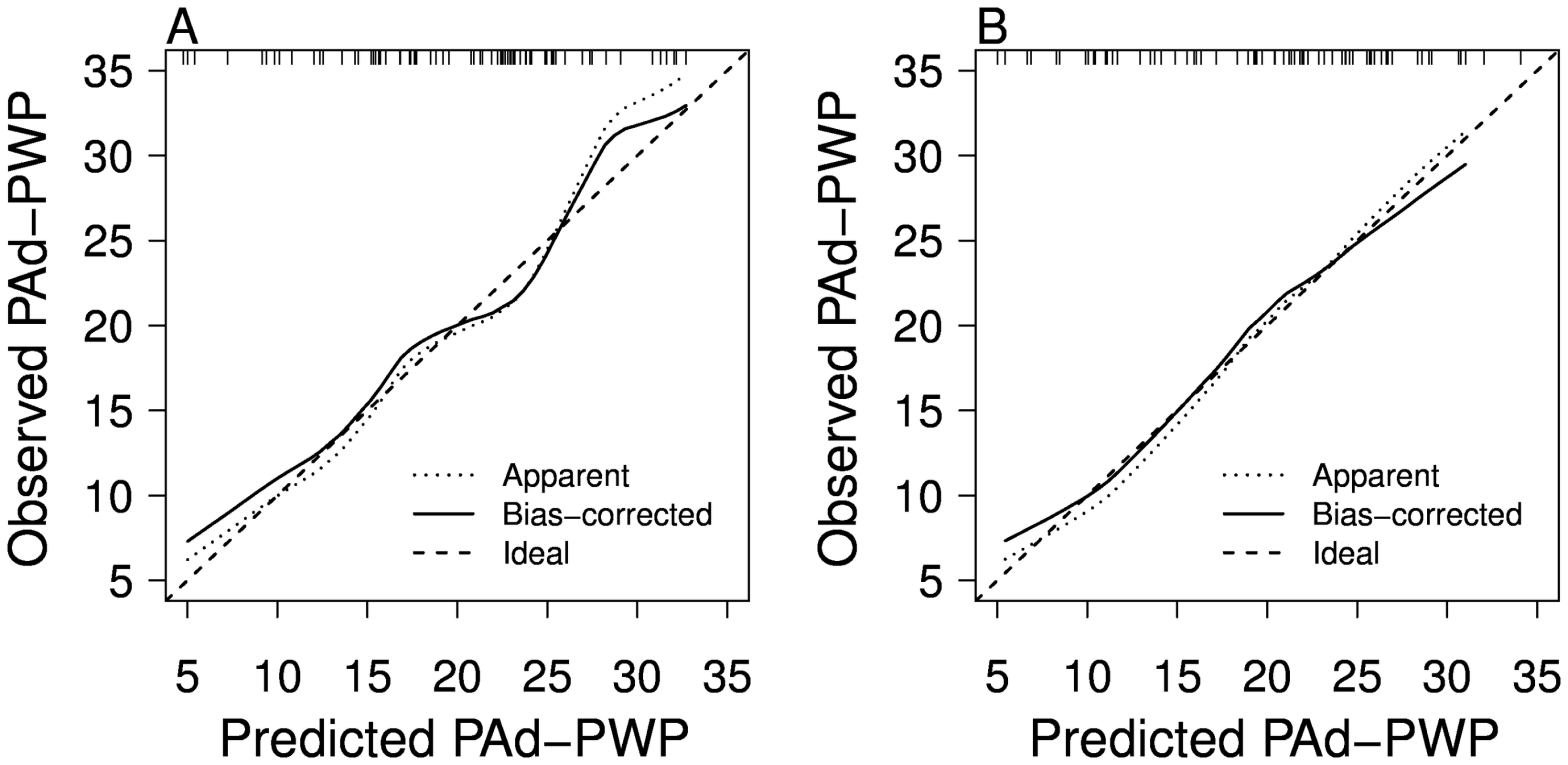
Calibration Model for the Prediction of Pulmonary Artery Diastolic to Wedge Gradient Using Clinical and Echocardiographic Variables. Panel A shows the prediction model calibration curve without inclusion of echocardiographic variables. Panel B shows the model with echocardiographic variables. Accuracy of the predicted PAd-PWP is improved (slope 0.89), especially in the range of PAd-PWP that represents a diagnostic dilemma (~ 5-15mmHg). PAd-PWP = pulmonary artery diastolic to wedge gradient.

Echocardiographic factors were more predictive than the clinical factors; we therefore chose the model with echocardiographic variables and risk factors to predict PAd-PWP. The proportion of variation explained by the model is R^2^ = 0.507, with adjusted R^2^ = 0.427. We used bootstrapping to validate and calibrate the model. The bias-corrected R^2^ was 0.35. The slope shrinkage factor was 0.89. Figure **2** depicts the model’s calibration curve, which plots the predicted vs. observed values. The mean absolute error was 0.59mmHg and the 0.9 quantile of the absolute error was 1.35mmHg.

As an example of practical clinical application, Table **6** shows the predicted PAd-PWP using the components of the final model for two patients, one with PAH and one with PVH, selected from our cohort.

**Table 6:** Application of Prediction Model to Two Patients in Cohort.

**Effect on PAd-PWP**	**Patient**		**Variable**	**Patient**	**Effect on PAd-PWP**	
-3.4			**Constant**		-3.4	
-3.0	52		**Age**	78	-4.5	
4.33	Female		**Sex**	Female	4.33	
10.6	29.5		**BMI**	31.9	11.4	
8.5	64		**Diastolic BP**	74	9.8	
-5.94	Yes		**Diabetes**	Yes	-5.94	
0	No		**Hypertension**	Yes	-6	
0	No		**Beta blocker use**	Yes	-1.89	
4.85	Yes		**RVH on EKG**	No	0	
11.4	4		**Echocardiographic Variables**	0	0	
**27.3mmHg**			**Predicted PAd-PWP**		**3.8mmHg**	
**Invasive Hemodynamics:**				**Invasive Hemodynamics**		
mPA = 67				mPA = 32		
mPWP = 12				mPWP = 22		
Pad = 41				PAd = 22		
**PAd-PWP = 29**				**PAd-PWP = 0**		
**Final Diagnosis: PAH**				**Final Diagnosis: PVH**		

BMI = body mass index; BP = blood pressure; EKG = electrocardiography; PAd-PWP = pulmonary artery diastolic to wedge gradient; PAH = pulmonary arterial hypertension; PVH = pulmonary venous hypertension; RVH = right ventricular hypertrophy

## Discussion

In this prospective study of new and returning patients referred for PH evaluation, we have shown that a combination of readily available clinical parameters and simple echocardiographic measurements is able to accurately predict the PAd-PWP gradient. We found that a combination of clinical risk factors and imaging parameters better predicted PAd-PWP than either group alone, which may explain some of the limitations of imaging-only models. Finally, we demonstrated that our model has good internal validity using a bootstrapping technique.

Both PAd-PWP and TPG were significantly different between the PAH and PVH cohorts. However, the mean value of TPG for the PVH group (15.7 mmHg) was elevated. Based on that value, according to current guidelines, the average PVH patient in our cohort may be considered by some physicians to have mixed etiology PH or even for PAH-specific therapy [19]. However, when the PAd-PWP is used, the mean for the PVH group (2.7 mmHg) is not elevated and suggests that vascular remodeling is not a major contributor to pulmonary artery pressure [10].

### Prediction Model Creation and Validation

We deliberately chose echocardiography variables that can easily be assessed in any patients with adequate image quality and avoided highly-quantitative or Doppler-based measurements. Quantitative measurements such as the tricuspid annular plane systolic excursion (TAPSE) and fractional area change (FAC) reflect RV function and predict outcomes in PAH but cannot differentiate PAH and PVH. Doppler parameters such as notching of the right ventricular outflow tract or pulmonary acceleration time (PAT) better reflect underlying physiology but are limited by subjective interpretation (notching mid- versus late systole) and vary based on acquisition technique (placement of pulse Doppler sample). Visual assessment should not be the only method for determining RV function, but is reliable when performed by an experienced reader [20]. RV dilation, hypertrophy, and paradoxical septal motion can be easily assessed from standard views.

The cluster of echocardiographic variables was by far the most influential element in the prediction model. The inclusion of several other pre-specified risk factors (age, sex, BMI, diastolic blood pressure, DM, HTN, beta blocker use and RVH on EKG) provided additional predictive value. Surprisingly, the clinical variables we chose (CAD, prior cardiac surgery, prior PCI, history of atrial fibrillation (AF) or flutter, and prior AF ablation) did not provide incremental value and were thus excluded from the final model. It is likely that the predictive value inherent in the clinical variables was accounted for in the risk factors included in the model.

Our model appears to be least accurate at the extremes of predicted PAd-PWP; however, in the range of PAd-PWP that presents a diagnostic dilemma, > 5mmHg to < 15mmHg, the model is highly accurate. Patients with predicted PAd-PWP > 15mmHg could be confidently assessed to be high risk for PAH and be referred for invasive hemodynamics to confirm the diagnosis and perform vasodilator testing. Conversely, patients with predicted PAd-PWP < 5mmHG are highly likely to have PVH and further diagnostic work-up could focus on underlying causes of elevated LAP and may not necessitate RHC.

### Non-Invasive Prediction Models

Several echocardiography-based prediction models to differentiate PAH and PVH have been described. Opotowski et al developed a prediction rule using left atrial dimension, presence or absence of systolic notching of the RVOT Doppler signal, PAT, and lateral mitral E/e’. The prediction rule performed quite well inpatients who clearly had hemodynamic evidence of PAH or PVH, but did not reliably differentiate patients with hemodynamic evidence of “mixed PH” (i.e. elevated PASP and PWP). The score presented is easy to use but may fail to accurately classify patients with ambiguous hemodynamics. Willens et al examined the ability of various LV diastolic parameters to differentiate PAH and PVH. The variables studied, especially E/e’ (AUC 0.97, 95% CI 0.88-0.99), performed very well. The mean TPG in the patients with PVH (13.8±7.2mmHg) was above the conventional cutoff of 12 whereas the PAd-PWP was essentially zero, again indicating the limitations of TPG and likely superiority of PAd-PWP as a discriminator between PAH and PVH [19]. Despite excellent performance, all the measurements studied require manual measurement and are angle-dependent, potentially limiting their practicality outside of academic centers where comprehensive Doppler examination may not be routine. Bonderman et al studied the ability of echocardiography, electrocardiography, and serum N-terminal brain natriuretic peptide to exclude PAH in a mixed-etiology cohort referred for PH evaluation [21]. The algorithm performed quite well at excluding pre-capillary PH (sensitivity 100%), but at the cost of decreased specificity (19%). In practice, the authors report that this algorithm would allow withholding RHC in only 9% of patients referred for PH evaluation.

### Clinical Relevance of Diastolic-to-Wedge Prediction Model

The treatment and prognosis of PAH and PVH differ dramatically, underscoring the need for accurate diagnosis. The ability to predict PAd-PWP gradient and thus differentiate PAH and PVH has a number of potential advantages over routine referral for invasive hemodynamics. RHC cannot practically be performed in the increasing number of patients with PH due to left heart disease. A model that accurately predicts the underlying physiology of PH would maximize identification of high-risk patients for PAH to select for confirmatory RHC. Patients predicted to have PVH would be considered for further or alternative diagnostic testing and therapy directed at left heart disease. Patients likely to have PVH based on clinical factors but with a predicted PAd-PWP > 7mmHg may have developed a reactive component or “out-of-proportion” PH. Our cohort included 2 such patients, both of whom were found to have very high PWP. Therefore, this model does not discriminate patients with “out-of-proportion” PH, but these patients should be referred for RHC as they may require evaluation by a specialist for appropriate classification of PH [3]. A retrospective study of over 2300 patients recently showed that PAd-PWP ≥ 7 in patients is associated with worse median survival compared to patients with PAd-PWP < 7 [22]. Finally, routine referral to RHC is costly and confers risk of bleeding, infection, and myocardial damage in addition to radiation exposure.

### Limitations

These findings are from a single center; an independent validation cohort would be needed to confirm the utility of this model. The number of patients with PVH is small because we included both new referrals and returning patients, most of whom have PAH. Therefore, our cohort is heavily skewed toward PAH patients which have influenced the accuracy of our prediction model. In addition, we excluded patients with LV systolic dysfunction. Our model incorporates more variables than some other prediction models, but all the variables we included are routinely collected as part of a clinical evaluation of PH. The determination of RV function was based on visual assessment without any confirmatory quantitative measures as recommended by the American Society of Echocardiography [17]. However, visual assessment of RV free wall motion abnormalities is accurate when performed by experienced readers and this present/absent approach is highly practical in non-academic centers [20]. The time from echocardiography to diagnostic catheterization in our study spanned up to 6 months. This gap reflects the obstacles inherent in clinical practice and any decline in RV function between echocardiography and catheterization would limit the predictive power of the model and bias to the null hypothesis.

## Conclusions

We have shown that a combination of clinical and simple echocardiographic variables can accurately predict the pulmonary artery diastolic pressure to wedge pressure gradient in patients with PAH, PVH, and NoPH. The PAd-PWP gradient is a better physiologic reflection of underlying etiology of PH compared to the TPG and reliably discriminated between PAH and PVH in our cohort. The model presented is most accurate in the range of PAd-PWP that may present a diagnostic dilemma (5-20mmHg) reinforcing its potential clinical usefulness. Our model requires further testing and independent validation, but may allow clinicians to be more parsimonious with referral for invasive testing in the work-up of pulmonary hypertension.
